# Cytotoxic T Cells Mediate Pathology and Metastasis in Cutaneous Leishmaniasis

**DOI:** 10.1371/journal.ppat.1003504

**Published:** 2013-07-18

**Authors:** Fernanda O. Novais, Lucas P. Carvalho, Joel W. Graff, Daniel P. Beiting, Gordon Ruthel, David S. Roos, Michael R. Betts, Michael H. Goldschmidt, Mary E. Wilson, Camila I. de Oliveira, Phillip Scott

**Affiliations:** 1 Department of Pathobiology, School of Veterinary Medicine, University of Pennsylvania, Philadelphia, Pennsylvania, United States of America; 2 Instituto Nacional de Ciência e Tecnologia de Doenças Tropicais-INCT-DT(CNPq/MCT), Serviço de Imunologia, Hospital Universitario Prof. Edgard Santos, Universidade Federal da Bahia Salvador, Bahia, Brazil; 3 Iowa City VA Medical Center, Iowa City, Iowa, United States of America; 4 Department of Biology, University of Pennsylvania, Philadelphia, Pennsylvania, United States of America; 5 Department of Microbiology, Perelman School of Medicine, University of Pennsylvania, Philadelphia, Pennsylvania, United States of America; 6 Centro de Pesquisas Gonçalo Moniz, Fundaçao Oswaldo Cruz, Salvador, Brazil; University of Texas Medical Branch, United States of America

## Abstract

Disease progression in response to infection can be strongly influenced by both pathogen burden and infection-induced immunopathology. While current therapeutics focus on augmenting protective immune responses, identifying therapeutics that reduce infection-induced immunopathology are clearly warranted. Despite the apparent protective role for murine CD8+ T cells following infection with the intracellular parasite *Leishmania*, CD8+ T cells have been paradoxically linked to immunopathological responses in human cutaneous leishmaniasis. Transcriptome analysis of lesions from *Leishmania braziliensis* patients revealed that genes associated with the cytolytic pathway are highly expressed and CD8+ T cells from lesions exhibited a cytolytic phenotype. To determine if CD8+ T cells play a causal role in disease, we turned to a murine model. These studies revealed that disease progression and metastasis in *L. braziliensis* infected mice was independent of parasite burden and was instead directly associated with the presence of CD8+ T cells. In mice with severe pathology, we visualized CD8+ T cell degranulation and lysis of *L. braziliensis* infected cells. Finally, in contrast to wild-type CD8+ T cells, perforin-deficient cells failed to induce disease. Thus, we show for the first time that cytolytic CD8+ T cells mediate immunopathology and drive the development of metastatic lesions in cutaneous leishmaniasis.

## Author Summary

Leishmaniasis is a parasitic disease where the host immune response plays an essential role in pathogenesis. However, the mechanisms promoting immunopathology in patients are still unclear. We performed gene expression profiling of skin lesions from cutaneous leishmaniasis patients and normal skin and the results demonstrated that the most expressed genes in leishmanial lesions were associated with the cytolytic pathway. Using both human samples and mouse models we showed that CD8+ T cells are cytolytic within leishmanial lesions and kill *Leishmania* infected target cells. We found that the CD8+ T cell cytolytic response was not protective, but rather promoted increased immunopathology, associated with enhanced recruitment of neutrophils to the site of infection. CD8+ T cells also promoted the development of metastatic lesions at distant skin sites. Together, our results clearly demonstrate that activation of CD8+ T cell cytolytic responses is detrimental to the host and that targeting this pathway could be a new approach to treat patients with leishmaniasis.

## Introduction

CD8+ T cells contribute to the control of pathogens by cytokine production, cytolytic activity or both. In the case of intracellular parasites, the production of IFN-γ by CD8+ T cells is protective, while in viral infections CD8+ T cells provide protection by inducing cytokine production and killing virally infected cells [1]. Nevertheless, these same CD8+ T cell effector functions can also promote increased pathology, and the presence of CD8+ T cells has been associated with increased pathology in several infectious and autoimmune diseases [2], [3], [4], [5], [6], [7], [8]. In some cases the pathology is believed to be associated with IFN-γ or IL-17 production, while in other situations cytolytic activity is linked with disease. Still, the mechanistic basis by which CD8+ T cells could potentially contribute to increased pathology is difficult to determine in humans.

Cutaneous leishmaniasis is one of many diseases where the outcome of the infection depends on both the extent of parasite elimination and the relative induction of potentially immunopathologic responses. A great deal is known about how leishmania parasites are eliminated. Thus, control of these intracellular parasites requires a CD4+ Th1 cell response, which leads to IFN-γ production that enhances the killing capacity of infected macrophages and dendritic cells [9], [10]. CD8+ T cells respond during infection and contribute to the control of *Leishmania* by producing IFN-γ, which not only activates macrophages to kill the parasites, but also promotes the differentiation of naïve T cells into Th1 cells [11], [12]. On the other hand, few studies have addressed how immunopathology develops in cutaneous leishmaniasis. Correlations with enhanced immunopathology and lower levels of IL-10 or IL-10 receptor expression have been observed in patients, but the unregulated responses that promote pathology are not defined [13], [14].

In patients infected with *L. braziliensis*, the number of CD8+ T cells increases in lesions as the disease worsens, and patients with mucosal disease–where metastatic lesions develop in the nasopharyngeal mucosa due to a destructive inflammatory response–have elevated numbers of cytotoxic cells in the blood [15], [16], [17]. Interestingly, it has also been reported that *L. major*-infected *Rag1−/−* mice reconstituted with CD8+ T cells develop much larger lesions than unreconstituted *Rag1−/−* mice [11]. Together, these observations implicate CD8+ T cells as inducers of pathology. As CD8+ T cells can produce IFN-γ in leishmaniasis, it is possible that an overproduction of IFN-γ promotes increased pathology. On the other hand, the severity of disease in patients infected with *L. braziliensis* is directly associated with increased numbers of granzyme expressing CD8+ T cells [15]. Thus, it remains to be determined whether CD8+ T cells are indeed pathogenic, and if so, whether they increase disease severity by cytokine production and/or enhanced cytolytic activity.

Defining the mechanisms that promote the immunopathology observed in cutaneous leishmaniasis is a critical first step in developing an approach to control the disease. Here, we define the pathologic role that CD8+ T cells play in *L. braziliensis* infections. We found that the most highly expressed genes in leishmanial lesions are associated with the lytic pathway and that CD8+ T cells within the lesions of *L. braziliensis* patients are functionally cytolytic. Using a murine model we found that CD8+ T cells contribute to increased lesion size following infection with *L. braziliensis* parasites. Strikingly, we found that the development of metastatic lesions was also promoted by the presence of CD8+ T cells. Mechanistically, we demonstrated that the pathology associated with unregulated CD8 function is not due to enhanced IFN-γ or IL-17 production, but rather is due to excessive perforin-dependent cytolytic activity by CD8+ T cells. Thus, our findings show for the first time that cytolytic CD8+ T cells are not only present during infection, but that they promote increased immunopathology and metastatic lesions in cutaneous leishmaniasis.

## Results

### CD8+ T cells from *L. braziliensis* patients exhibit increased cytolytic activity

**Figure 1:**
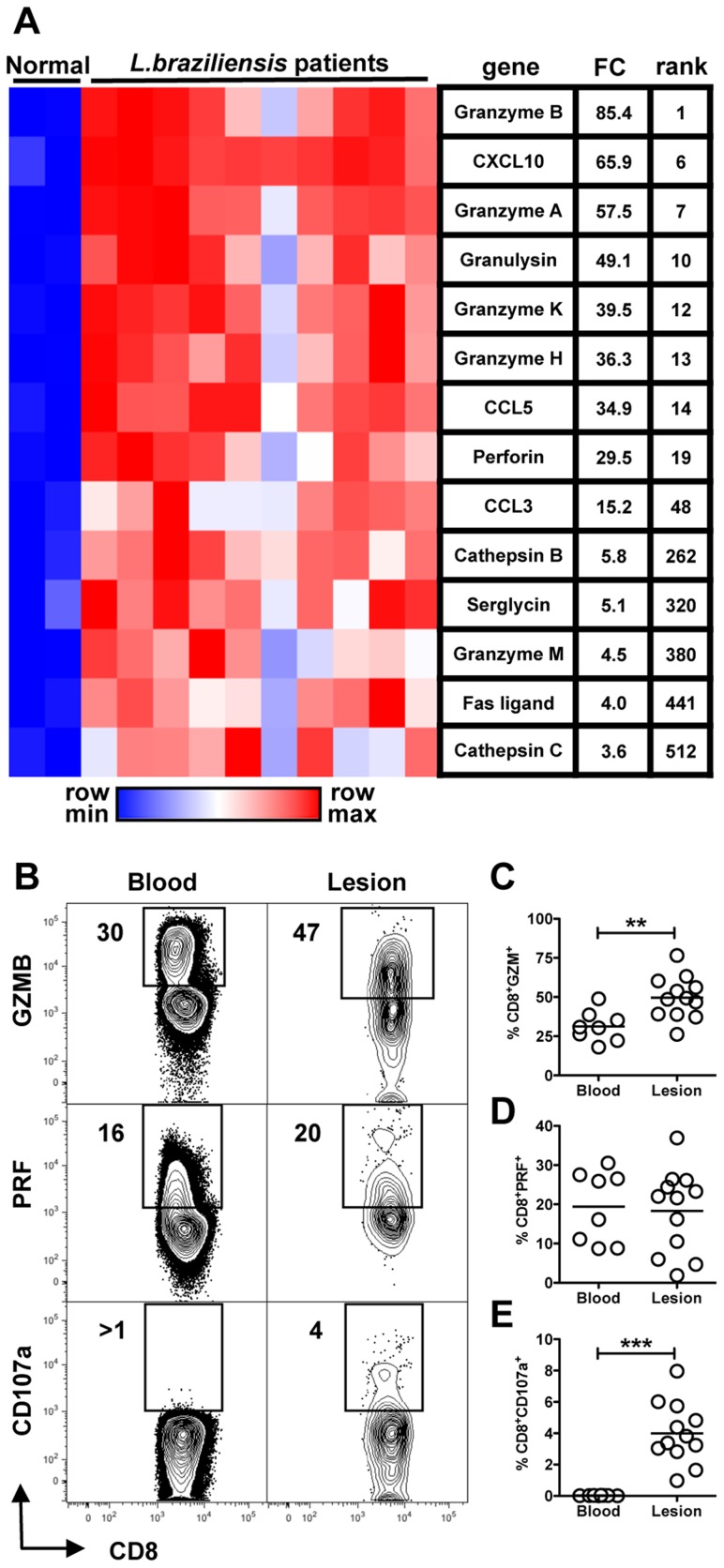
Cytolytic profile of CD8+ T cells in lesions from *L. braziliensis* infected patients. (A) Heatmap showing induction of key mediators of cytolysis from microarray profiling of ten human lesion biopsies and two normal skin biopsies. Average fold change (FC) for each gene in lesion samples, relative to normal skin controls, is shown, as is the rank for this fold change within the entire expression data set. (B) PBMCs and cells isolated from lesions obtained from *L. braziliensis* patients were incubated with anti-CD107a. Depicted are representative (A) contour plots and scatter plots of (C) GZMB, (D) PRF and (E) CD107a expression by CD8+ T cells (pregated on Singlets/CD3+/CD8+). Data from patients [8 (blood) and 12 (biopsy)] obtained in two independent experiments are shown. **p<0.01; ***p<0.001.

To better understand the local immune environment during human leishmaniasis and the extent to which a cytolytic program is associated with the infection, we carried out whole genome expression profiling of lesions from patients infected with *L. braziliensis*. Over 500 genes were expressed ≥3-fold (*p*≤0.05) in lesions compared to normal skin from uninfected donors (not shown). Strikingly, many known components of cytolytic granules were found amongst the most strongly expressed genes in lesions [18] (Fig. 1A). For example, granzyme B, granzyme A and granulysin, a pore-forming molecule found in the granules of human CTLs and NK cells, were the first, seventh and tenth genes most strongly expressed genes overall in lesions, respectively. Moreover, gene set enrichment analysis of the entire expression data set revealed a significant enrichment for KEGG pathways involved in NK cell mediated cytotoxicity, graft-versus-host diseases and allograft rejection (not shown), all of which involve cytolysis [19].

Next we measured the protein levels of granzyme B and perforin in CD8+ T cells recovered from the peripheral blood or from lesions of *L. braziliensis* infected patients (Fig. 1B). More CD8+ T cells obtained from lesions expressed granzyme B (Fig. 1C) in comparison to cells from the blood, and both populations contained cells expressing perforin (Fig. 1D). These data suggest that both skin and peripheral CD8+ T cells have the capacity to degranulate. To determine if in fact these cells were degranulating, we assessed their surface expression of CD107a (Fig. 1B, lower panels). CD107a is a lysosomal membrane glycoprotein (also known as Lamp1) present in CD8+ T cell granules. During degranulation, this molecule is transiently exposed on the cell surface and thus is a marker for the release of cytotoxic granules by CD8+ T cells [20]. We found a significant increase in CD8+ T cells expressing surface CD107a from lesions, while CD8+ T cells from the blood failed to express CD107a (Fig. 1E). These results not only confirm previous studies showing that CD8+ T cells express granzyme in leishmaniasis [15], but also extend these findings to show that the CD8+ T cells are cytolytically active within human leishmanial lesions.

### CD8+ T cells induce pathology in *L. braziliensis* infected mice

**Figure 2:**
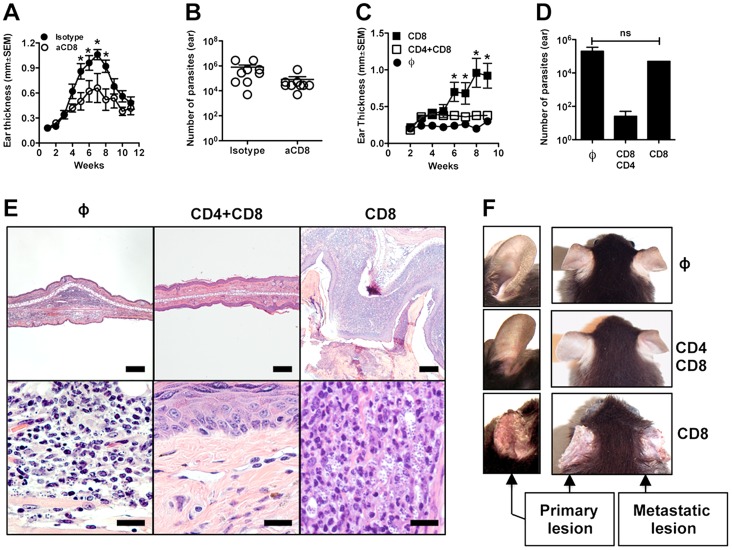
CD8+ T cells induce pathology during *L. braziliensis* infection. (A) BALB/c mice were infected with *L. braziliensis* in the ear, and the course of infection monitored, and (B) parasite burden assessed in the lesions at 5 weeks. *Rag1−/−* mice were infected with *L. braziliensis* in the ear, and reconstituted with either CD8+ T cells, or CD8+ and CD4+ T cells, or did not receive cells and (C) ear thickness at the site of infection assessed. At 7 weeks post infection mice were euthanized and shown are (D) the parasite burden in the lesions and (E) histological comparison of lesions by H&E staining. Scale bar represents 200 µm for lower magnification (top row) and 20 µm for higher magnification (bottom row). (F) Representative front images and back images (showing metastasis) of leishmanial lesions at 6 weeks post infection. Representative data from one of three or more independent experiments (n = 5) with similar results are presented. ns, non-significant. *p<0.05.

To determine if our results from *L. braziliensis* patients could be mechanistically dissected using animal models we first asked if depletion of CD8+ T cells would affect lesion development in BALB/c mice. In contrast to *L. major*, *L. braziliensis* infections in BALB/c mice results in the development of an ulcerated lesion that eventually resolves [21]. Thus, BALB/c mice infected with 10^5^ *L. braziliensis* developed a substantial lesion that healed spontaneously when treated with control isotype antibody (Fig. 2A, closed circles). In contrast, mice depleted of CD8+ T cells with an anti-CD8-specific antibody developed substantially smaller lesions (Fig. 2A, open circles), suggesting that CD8+ T cells contribute to lesion size. The relative decrease in lesion size in CD8 depleted mice was not due to an alteration in parasite number (Fig. 2B), indicating that the change in lesion size was due primarily to differences in the inflammatory response. Thus, these data indicate that CD8+ T cells contribute to the inflammatory response following *L. braziliensis* infection in BALB/c mice.

Two factors seem to be associated with immunopathology in *L. braziliensis* patients: an increase in CD8+ T cells recruited to the lesions and a decrease in immunoregulatory cytokines. To test if CD8+ T cells could directly enhance disease, *L. braziliensis* infected *Rag1*−/− mice were reconstituted with naive CD8+ T cells (RAG+CD8) or CD8+ and CD4+ T cells (RAG+CD4+CD8), at a 1∶1 ratio, and the course of infection was followed. Unreconstituted *Rag1*−/− mice infected with *L. braziliensis* developed minimal lesions. Thus, similar to *L. major* or *L. amazonensis* [11], [22], lesion development with *L. braziliensis* likely depends upon generation of a T cell-dependent inflammatory response. As expected, RAG+CD4+CD8 mice developed small nodules that resolved within 7 weeks following infection (Fig. 2C). In contrast, transfer of CD8+ T cells alone to *Rag1*−/− mice led to the development of an uncontrolled lesion (Fig. 2C). To determine if the increased pathology observed in RAG+CD8 mice was due to uncontrolled parasite growth, parasite loads were assessed within the lesions. We found that *Rag1*−/− mice and RAG+CD8 mice had similar numbers of parasites at the infection site, in spite of the disparity in lesion size observed in these animals, while transfer of CD4+ and CD8+ T cells into *Rag1*−/− mice led to significantly better control of the parasite in the infected ear (Fig. 2D). Thus, the exacerbated lesion development in RAG+CD8 mice compared with *Rag1*−/− mice is due to CD8+ T cell mediated pathology rather than differences in the number of parasites in the ear.

Most notably, the enhanced lesion size in RAG+CD8 mice was accompanied by a rampant immunopathologic response. By 5 weeks post infection we observed destruction of the infected ear in RAG+CD8 mice, but minimal pathology in either *Rag1*−/− or RAG+CD4+CD8 mice (Fig. 2E). This destruction was accompanied by infiltration of many more CD8+ T cells than in *Rag1*−/− mice that received both CD4+ and CD8+ T cells (data not shown). Histologically, we could observe at lower magnification the substantial differences in lesion thickness in mice without T cells and RAG+CD4+CD8 or RAG+CD8 mice. Higher magnification showed that lesions from *Rag1*−/− mice were composed of infected macrophages and granulocytes (Fig. 2E). At this time point, lesions from RAG+CD4+CD8 mice were healing and exhibited a mild dermal lymphocytic infiltrate accompanied by epidermal hyperplasia and spongiosis with few leishmania organisms (Fig. 2E). In contrast, the lesions from RAG+CD8 mice showed a dramatic cellular infiltration consisting of lymphocytes, granulocytes and many highly infected cells (Fig. 2E). Moreover, the epidermis in these lesions exhibited substantial hyperplasia and areas of ulceration, and the severe inflammatory response in the dermis led to alterations in the cartilage. Using a pathology score that goes from 0 to 5, where 0 is mild and 5 is severe disease, at 7 weeks post-infection RAG+CD8 mice had the most severe disease (5) followed by *Rag1−/−* mice with moderate disease (2) and RAG+CD4+CD8 mice with no disease (0). We also characterized the myeloid cell composition present in lesions from RAG+CD8 mice, and found that a majority of the myeloid cells within lesions were neutrophils. (Fig. S1). Together, these observations illustrate the critical role of the inflammatory response as the main factor driving lesion development, further highlighting the importance of identifying the mechanisms that control pathology in cutaneous leishmaniasis.

An additional unexpected result observed in RAG+CD8 mice was the development of metastatic lesions. This was particularly notable in the contralateral ear, which developed gross pathology indistinguishable from the primary lesion (Fig. 2F). In addition to the contralateral ear, we observed lesions at other skin sites, including the nose, tail, and footpad. We were able to culture parasites from these regions, confirming the spread of parasites to these additional skin sites (data not shown). In contrast, we did not observe metastatic lesions in *Rag1*−/− or RAG+CD4+CD8 mice (Fig. 2F). Thus, the development of both primary and metastatic lesions in *Rag1*−/− mice was dependent upon CD8+ T cells.

### CD8+ T cells within lesions express IFN-γ, IL-17 and high levels of granzyme B

**Figure 3:**
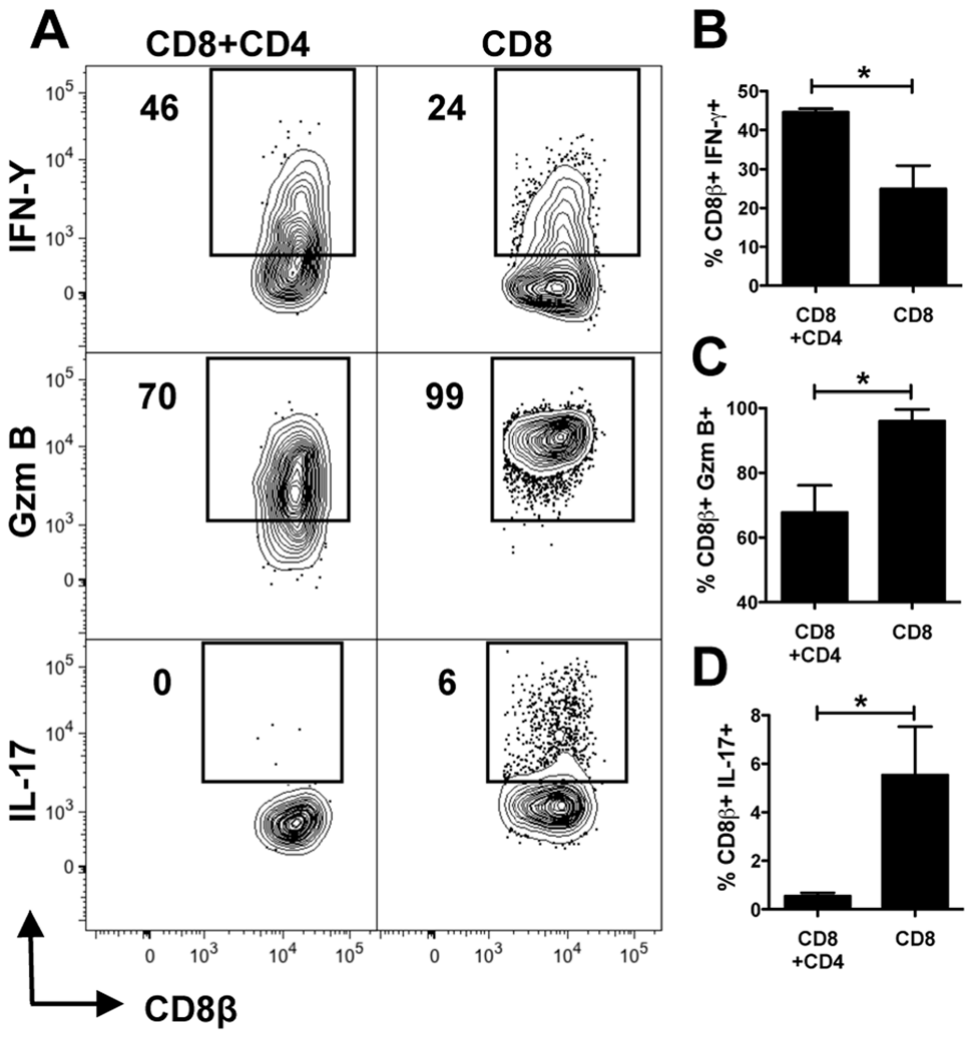
CD8+ T cells from lesions with severe pathology produce IFN-γ, granzyme B and IL-17. *Rag1−/−* mice were infected with *L. braziliensis* in the ear, and reconstituted with either CD8+ T cells, or CD8+ and CD4+ T cells. At 7 weeks post infection mice were euthanized and cells from the lesions were stained for flow cytometry. Depicted are representative (A) contour plots and bar graphs of intracellular (B) IFN-γ, (C) GzmB and (D) IL-17 (pregated on Live/Singlets/CD45+/CD8β+ cells). Bar graphs are representative data from one of three independent experiments (n = 5) with similar results. *p<0.05.

To characterize the functions of the CD8+ T cells transferred in the presence and absence of CD4+ T cells, cells from lesions of *L. braziliensis* infected reconstituted *Rag1*−/− mice were assessed for IFN-γ, IL-17 and granzyme B by flow cytometry (Fig. 3A). A higher percentage of CD8+ T cells made IFN-γ when these cells were transferred together with CD4+ T cells, although CD8+ T cells made IFN-γ in the absence of CD4+ T cells (Fig. 3B). In contrast, CD8+ T cells from RAG+CD8 mice produced significantly more granzyme B in the absence of CD4+ T cells (Fig. 3C). Finally, although only a small percentage of CD8+ T cells from RAG+CD8 mice produced IL-17, IL-17 production was completely abrogated by the presence of CD4+ T cells in RAG+CD4+CD8 mice (Fig. 3D). Overall, these results suggest that CD8+ T cells could be mediating increased pathology due to cytolytic activity (indicated by high levels of granzyme B), IL-17, or IFN-γ, and indicate that CD4+ T cells may regulate these responses.

### CD8+ T cells degranulate and kill *L. braziliensis* infected cells

**Figure 4:**
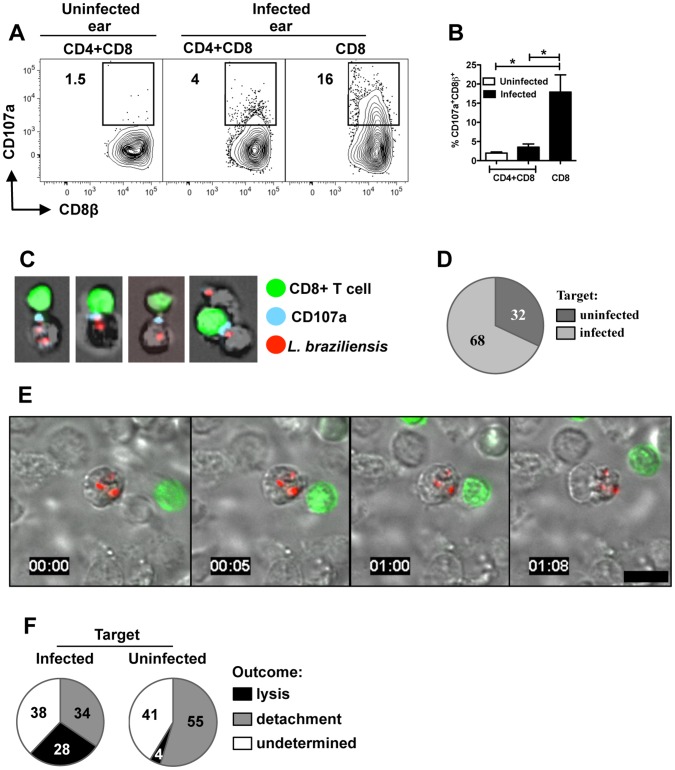
CD8+ T cells degranulate and induce killing of *L. braziliensis* infected target cells. (A,B) *Rag1−/−* mice were infected with *L. braziliensis* in the ear, and reconstituted with either CD8+ T cells, or CD8+ and CD4+ T cells. Four to six weeks post infection cells isolated from uninfected and infected ears were incubated with anti-CD107a. Depicted are representative (A) contour plots and (B) bar graph of CD107a expression by CD8+ T cells (pregated on Live/Singlets/CD45+/CD8β+ cells). The bar graph represents data from five independent experiments. *p<0.05. (C) *Rag1−/−* mice were infected with mCherry expressing *L. braziliensis* in the ear, and reconstituted with eGFP CD8+ T cells. Six weeks post infection cells isolated from infected ears were incubated with anti-CD107a and acquired by ImageStream. Depicted are four representative images showing CD107a expression at the interface between CD8+ T cells and *L. braziliensis* infected targets. (D) Pie chart with the percentage of eGFP+ CD8+ T cells in contact with infected and uninfected target cells expressing CD107a as assessed by ImageStream. (E) Live-cell imaging of ear cells from *Rag1−/−* mice infected with mCherry expressing *L. braziliensis* and reconstituted with eGFP CD8+ T cells four to eight weeks post infection. Numbers represent time in hours∶minutes and scale bar 10 µm. (F) Pie charts with analysis of the outcome of CD8+ T cell and target cell interaction. Data from 63 live-cell imaging fields from 6 independent infection experiments.

As we observed expression of genes associated with cytolysis in leishmanial lesions, we first assessed if the immunopathology observed in RAG+CD8 mice was related to cytolytic activity. Cells were obtained 5 weeks after infection of RAG+CD4+CD8 or RAG+CD8 mice, and were stained for CD107a expression directly ex vivo. In RAG+CD4+CD8 mice analysis of CD107a expression showed a small percentage of degranulating CD8+ T cells at the infection site (Fig. 4A, 4B). On the other hand, a high percentage of CD8+ T cells from lesions of RAG+CD8 mice expressed surface CD107a (Fig. 4A, 4B).

To confirm that CD107a expression was indicative of degranulation, we sought to visualize CD107a at the interface between CD8+ T cells and infected cells. For these experiments, eGFP+ CD8+ T cells were transferred into *Rag1*−/− mice that were subsequently infected with *L. braziliensis* parasites expressing mCherry. Cells from lesions taken 5 weeks after infection were incubated for 1 hour in the presence of anti-CD107a monoclonal antibody and then run on an ImageStream flow cytometer. We observed the presence of CD107a (blue) at the synapse between CD8+ T cells (green) and *L. braziliensis* infected target cells (red) providing further support that CD8+ T cells from RAG+CD8 mice were degranulating (Fig. 4C). Analysis of the total doublets that contained eGFP+ CD8+ T cells showed that surface expression of CD107a on CD8+ T cells was more frequent when CD8+ T cells were in contact with infected in comparison to uninfected target cells (Fig. 4D).

Finally, to directly show that CD8+ T cells from RAG+CD8 mice induce infected cell death, we visualized the interactions between CD8+ T cells and infected cells using a spinning disk confocal microscope. As above, mice were infected with mCherry *L. braziliensis* parasites and reconstituted with GFP+ CD8+ T cells, and after 5 weeks cells from the lesions were isolated and immediately visualized. We observed several different types of interactions between CD8+ T cells and infected cells. In some cases, T cells moved around the surface of infected cells and ultimately detached from the target cell (Video S1). We also detected stable conjugates between infected cells and CD8+ T cells that, after an average of 25 minutes (ranging from 10 to 60 minutes), led to the formation of membrane blebs on the target cell, but with no visible damage to intracellular parasites. This was followed by immediate detachment (Figure 4E; Video S2) in most cases, while in some cases the CD8+ T cells remained in contact for several minutes after the target cell underwent apoptosis (Video S3). Analysis of 63 movies obtained from 6 individual experimental infections showed that infected cells were killed more frequently than uninfected cells (Fig. 4F), even though the number of uninfected cells was much greater in these preparations (data not shown). Due to the fact that cells can leave the imaging field, we could not always determine the outcome of CD8+ T cell-target interactions (undetermined). Thus, we expect that our analysis may have underestimated the number of times CD8+ T cell interactions with infected cells led to target cell killing.

### The pathology induced by CD8+ T cells requires antigen recognition

**Figure 5:**
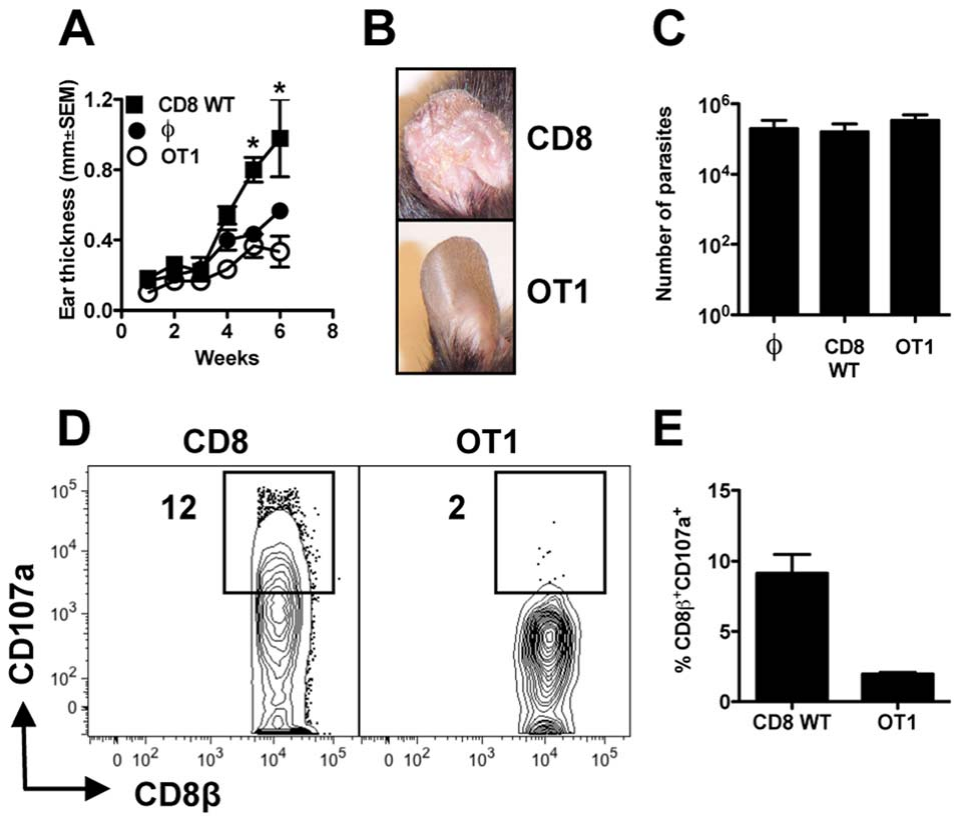
CD8+ T cell induced pathology requires specificity. *Rag1−/−* mice were infected with *L. braziliensis* in the ear, and reconstituted with either WT or OT1 CD8+ T cells. (A) Ear thickness was evaluated at the site of infection. (B) Representative images of leishmanial lesions at 6 weeks post infection. At 7 weeks post infection mice were euthanized and shown are: (C) parasite burden in the lesions; (D) contour plots and (E) bar graph of CD107a expression by CD8+ T cells (pregated on: Live/Singlets/CD45+/CD8β+ cells). Graphs are representative data from one of two independent experiments (n = 3) with similar results. *p<0.05.

Because CD8+ T cells preferentially lysed *L. braziliensis* infected cells, we hypothesized that this was a specific CD8+ T cell interaction. To test this, we transferred CD8+ T cells that would not recognize *L. braziliensis* (transgenic OT1 cells) or wild-type (WT) CD8+ T cells to *Rag1*−/− mice and infected them with *L. braziliensis* parasites. Confirming data shown in Fig. 2C, large lesions were detected in mice that received WT CD8+ T cells (Fig. 5A). In contrast, mice that received OT1 cells exhibited small lesions and no evidence of severe pathology (Fig. 5A and 5B). We found no differences in the number of parasites detected in the lesions of mice reconstituted with WT CD8+ T cells and OT1 cells, providing further evidence that the pathology observed in the RAG+CD8 mice was unrelated to the number of parasites observed in the lesions (Fig. 5C). Finally, we measured OT1 cell degranulation in the lesions of *L. braziliensis* infected *Rag1*−/− mice by assessing CD107a expression. No CD107a expression by the OT1 cells was observed, while a significant percentage of WT CD8+ T cells were expressing CD107a (Fig. 5D, 5E).

### *Prf1−/−* CD8+ T cells are unable to induce pathology in *L. braziliensis Rag1*−/− mice

**Figure 6:**
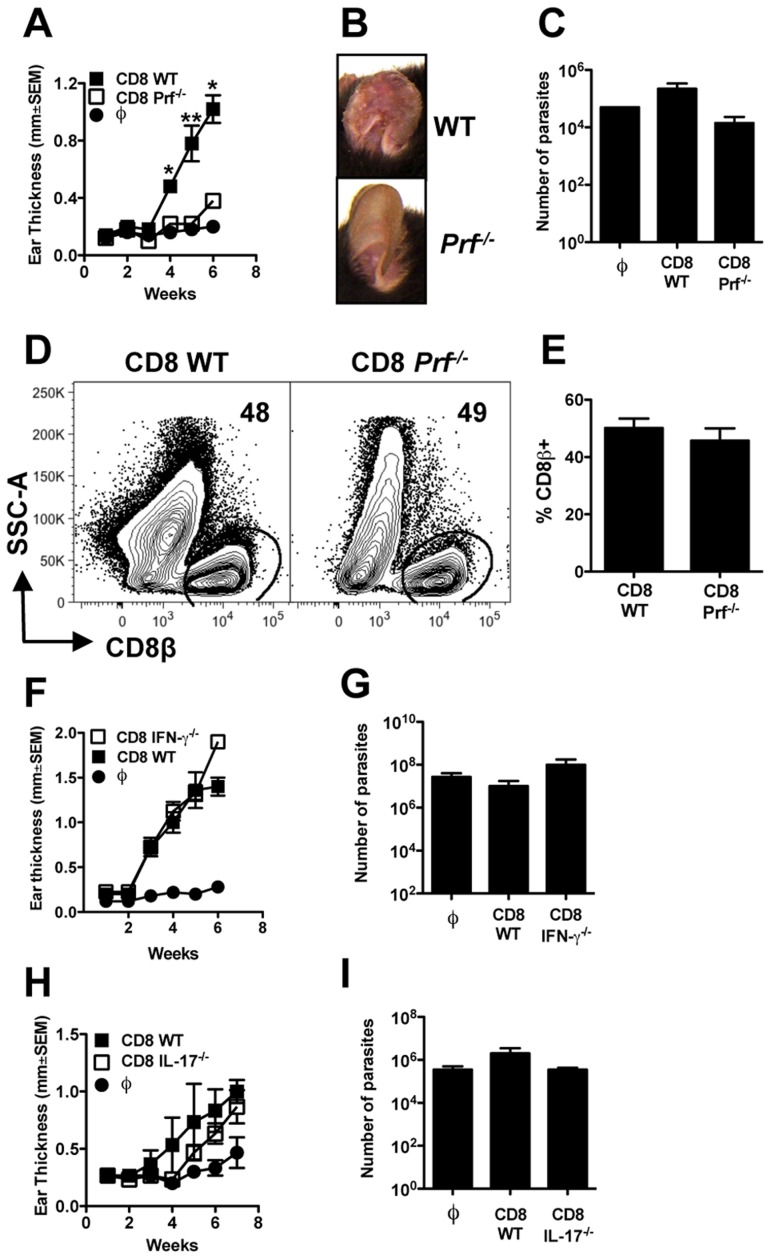
Perforin is required for CD8+ T cell induced pathology in *L. *braziliensis infection. *Rag1−/−* mice were infected with *L. braziliensis* in the ear, and reconstituted with either WT or *Prf−/−* CD8+ T cells. (A) Ear thickness at the site of infection. (B) Representative images of leishmanial lesions at 5 weeks post infection. At 7 weeks post infection mice were euthanized and shown are: (C) parasite burden in the lesions; representative (D) contour plots and (E) bar graph of CD8+ T cell in the lesion (pregated on: Live/Singlets/CD45+/CD8β+ cells). Graphs are representative data from one of three independent experiments (n = 5). *Rag1−/−* mice were infected with *L. braziliensis* in the ear, and reconstituted with either WT, IFN-γ−/−, or IL-17−/− CD8+ T cells. (F,H) Ear thickness at the site of infection; (G,I) Parasite burden in the lesions at 7 weeks post infection. Data from one (F,G) experiment or one representative experiment of two (H,I) (n = 5) are presented. *p<0.05.

Although the induction of apoptosis by CTLs is often associated with minimal inflammation, cell death can also promote extensive inflammatory responses [23]. To directly assess if CD8+ T cell-mediated pathology is related to their cytolytic capacity, we next determined if CD8+ T cells required perforin to mediate immunopathology in RAG+CD8 mice. *Rag1*−/− mice received either *Prf1*−/− or WT CD8+ T cells and were infected with *L. braziliensis*. While WT CD8+ T cells promoted increased pathology, perforin deficient CD8+ T cells failed to promote lesion development (Fig. 6A) or severe pathology (Fig. 6B). Moreover, in contrast to WT CD8+ T cells, mice that received *Prf1*−/− T cells failed to develop metastatic lesions (data not shown). This difference in lesion development was independent of the number of parasites in the primary lesions as the parasite load was the same in mice that received *Prf1*−/− and WT CD8+ T cells (Fig. 6C). The absence of severe disease in mice that received *Prf1*−/− CD8+ T cells was not due to differences in the capacity of CD8+ T cells from *Prf1*−/− mice to be recruited to the infected skin, since both groups had similar percentages of CD8+ T cells present in the skin (Fig. 6D, 6E).

We also tested if IFN-γ or IL-17 were required for immunopathology induced by CD8+ T cells. To do so, we transferred CD8+ T cells deficient in either IFN-γ or IL-17 and both groups of mice developed lesions similar to WT CD8+ T cells, with similar numbers of parasites, suggesting that these cytokines are not required for lesion development (Fig. 6F–I). Taking all of our results together, we conclude that perforin-dependent cytolytic activity is the main mechanism by which CD8+ T cells promote disease. Moreover, we have discovered a previously unappreciated role for CD8+ T cells in promoting the development of metastatic lesions in cutaneous leishmaniasis.

## Discussion

*Leishmania* are intracellular parasites that infect and multiply within myeloid-lineage cells, such as macrophages and DCs. They are transmitted to humans by sand flies and more than 10 different species can cause disease in humans. Cutaneous leishmaniasis is the most common clinical form of leishmaniasis and it is estimated that 1.5 million new cases of cutaneous leishmaniasis occur annually and 12 million individuals are exposed to infection worldwide [24]. The severity of cutaneous disease depends on both the extent of parasite replication and the relative induction of immunopathologic responses. For example, disease induced by *Leishmania braziliensis*, the leading causal agent of leishmaniasis in South America, has several clinical manifestations, all of which are associated with significant immunopathologic responses. The factors that mediate these immunopathologic responses are poorly defined. Here, by combining clinical data from patients with results from our experimental *L. braziliensis* infection, we have identified CD8+ T cell-mediated cytotoxicity as a major contributor to increased pathology in cutaneous leishmaniasis.

While CD4+ Th1 cells are critical for controlling *Leishmania*, CD8+ T cells also play a protective role, since mice lacking CD8+ T cells exhibit increased susceptibility to *L. major* [11], [12], [25]. Moreover, in several experimental vaccine models CD8+ T cells contribute to immunity [26], [27], [28], [29], [30], [31]. In contrast, we found that depletion of CD8+ T cells in *L. braziliensis* infected mice decreases inflammation. It is currently unclear why CD8+ T cell depletion has different effects in *L. major* and *L. braziliensis* infected mice. However, CD8+ T cell derived IFN-γ blocks the development of a Th2 response in *L. major* infected mice [12], and the apparent lack of Th2 induction in *L. braziliensis* infection may contribute to these differential effects [21], [32]. Nevertheless, our findings indicate that CD8+ T cells can promote increased disease. Although this may seem paradoxical, our current results suggest that a bifurcation in CD8+ T cell effector function provides a potential explanation. Specifically, CD8+ T cells provide protection by releasing IFN-γ, thereby activating macrophages to kill the parasites, and promoting a stronger CD4+ Th1 response in response to *L. major* infections [12]. In contrast, our current results with *L. braziliensis* suggest that cytolytic function, rather than IFN-γ production, promotes increased pathology. These findings are consistent with our own observations, as well as others, that in *L. braziliensis* patients there is a direct correlation between granzyme expressing CD8+ T cells in lesions and disease severity [15], [33]. While we found that perforin-expressing CD8+ T cells were required for this pathology, we cannot exclude a role for NK cells. Indeed, NK cells have been linked with pathology in mucocutaneous leishmaniasis patients [17] and NK cells were present in the lesions of *L. braziliensis* infected RAG+CD8 mice (data not shown). Similarly, we cannot exclude a role for CD8 derived chemokines in promoting infiltration of inflammatory cells into these lesions [34]. Taken together, the human studies suggest that CD8+ T cell cytolytic activity is pathologic, and using a mouse model we are the first to show conclusively that indeed a dysregulated CD8+ T cell response promotes perforin-dependent immunopathology in cutaneous leishmaniasis. Therefore, defining mechanisms that control CD8+ T cell function, including regulation by factors present within the cytokine milieu [35], will be a crucial step in identifying therapeutic targets to treat immunopathologic sequelae.

Surprisingly, the role of CTLs in cutaneous leishmaniasis has not been extensively explored. Nevertheless, studies have shown that CD8+ T cell lines or clones can be lytic for *Leishmania* infected cells [17], [34], [36], [37], [38], [39], [40]. Here, we extend those studies and show the ability of effector CD8+ T cells from infected mice to kill naturally infected targets. We were able to visualize for the first time CD8+ T cells lysing *L. braziliensis* infected targets and characterize the interactions that preceded the lytic event. In this regard, we found that CD8+ T cells bind target cells for an average of 25 minutes before target cell lysis, similar to what is seen for CD8+ T cell induced apoptosis of peptide pulsed B cells [41]. This killing appeared to be specific, since OT1 T cells–which cannot recognize leishmanial antigens–failed to degranulate or induce pathology in *L. braziliensis* infected mice. Additionally, while almost 40% of the interactions between CD8+ T cells and infected cells resulted in target cell lysis, a much smaller fraction (∼4%) of interactions between CD8+ T cells and uninfected cells resulted in apoptosis. Although the mechanisms promoting killing of uninfected targets was not explored, these targets may have internalized leishmanial antigens or phagocytosed infected apoptotic cells, resulting in cross presentation [42]. Alternatively, the killing could be antigen-independent, as occurs when stressed cells express ligands recognized by NKG2D on CD8+ T cells [43]. Finally, it remains to be determined whether parasites are killed when the target cell is killed. We were unable to determine the fate of the parasites following killing of their host cell, primarily because dying cells did not remain in the field of focus. However, since parasite numbers were similar in *Rag1*−/− and RAG+CD8 mice, it seems unlikely that the parasites are killed, although we cannot disregard the possibility that in an immunocompetent mouse the parasites might be killed. Consistent with this view, a previous study with a CTL clone indicated that parasites survive after their host cell is killed by CD8+ T cells [40].

CD8+ T cells are best known for their ability to protect against viral infections by lysing virus-infected targets. However, a pathologic role for CD8+ T cells has also been observed. For example, virally induced myocarditis is prevented in mice deficient in perforin [5]. Similarly, experimental cerebral malaria is mediated by CD8 CTLs [6], [7]. Perhaps most analogous to the pathologic CTL responses that we observe in leishmaniasis are data from experimental *Trypanosoma cruzi* infections in which CD8+ T cell responses that have traditionally been considered protective have more recently been linked to pathology [8], [44]. Such a finding is again consistent with a bifurcation in CD8+ T cell function in which IFN-γ producing CD8+ T cells are protective, while perforin expressing CD8+ T cells mediate increased pathology in the heart [8]. Leishmania infection is a good model to understand this duality of CD8+ T cell function in vivo, since CD8+ T cells are protective in visceral leishmaniasis [26], [45], [46], [47], whereas we now understand that this is the opposite for *L. braziliensis*. Understanding what mechanisms drive CD8+ T cells to become pathogenic or protective is an important goal to design new treatments and is now under investigation in our lab. At present, it is unclear why CTLs induce pathology in cutaneous leishmaniasis, since apoptosis is primarily thought to drive anti-inflammatory responses [48]. However, apoptotic cells can undergo secondary necrosis if not rapidly cleared by phagocytes. In secondary necrosis the integrity of the plasma membrane is lost and intracellular constituents of the cell are released, increasing the inflammatory response [23], and providing positive feedback to increase the cytolytic activity of CD8+ T cells [49], [50].

In several clinical forms of leishmaniasis, parasites metastasize to distant cutaneous sites. In mucosal leishmaniasis, metastatic lesions develop in the nasopharyngeal region, which leads to substantial morbidity [16], while in disseminated leishmaniasis individual nodules develop at multiple sites in the skin [51], [52]. It has been suggested that an RNA virus present in some South American strains of *Leishmania* enhances the immune response, potentially promoting metastasis [53]. However, this is not a universal finding, as parasite strains associated with metastatic disease do not all contain this virus (the strain that we have used here does not contain the virus). Nevertheless, the association of enhanced immune responses with metastasis is consistent with our results, and suggests that dysregulated immune responses play an essential role in this process. Although the mechanistic basis for immune-mediated metastasis is unclear, the immune system may simply be responding to disseminated parasites, thereby inducing the inflammatory response required for lesion development at distal sites. Alternatively, the immune response itself may contribute to parasite spread. One attractive possibility is that CTL killing of infected cells enhances the release of parasites, allowing them to metastasize more efficiently to distant skin sites.

The relative decrease in immunopathology in mice reconstituted with both CD4+ and CD8+ T cells suggests that CD4+ T cells control the pathogenicity of CD8+ T cells. This may be, in part, by controlling the number of CD8+ T cells or the number of parasites within the lesion. However, we also found that CD8+ T cells transferred in the absence of CD4+ T cells expressed relatively higher levels of GrzB and IL-17, and less IFN-y, than did CD8+ T cells co-transferred with CD4+ T cells. We predict that this difference is due to the absence of regulatory T cells (Tregs) since in tumor models Tregs control the cytotoxicity of CD8+ T cells in a TGF-β dependent manner [41], [54], and in leishmaniasis both CD4+ Th1 cells and CD4+ T regulatory cells (Treg) dampen the immune response [55], [56], [57]. While we have not yet determined how CD4+ T cells control the pathologic CD8+ T cell response, we believe that the ratio of CD4+ and CD8+ T cells may be a critical determinant in disease outcome, since we found that *Rag1*−/− mice developed disease even in the presence of CD4+ T cells, as long as they are in relatively low numbers (data not shown). This result is consistent with the observed change in ratio of CD4+ and CD8+ T cells present in lesions as disease progresses in human patients [15]. Thus, we suggest that an analysis of the CD4∶CD8 ratio in biopsies taken for diagnostic purposes might be useful in predicting disease outcome.

In summary, our results define a new role for CD8+ T cells in leishmaniasis. While CD8+ T cells have previously been thought of as primarily protective, our results demonstrate that they can mediate severe pathologic responses. This finding makes it essential that more effort is directed at delineating the factors that determine CD8+ T cell effector function during leishmaniasis, since such information is critical in considering therapies or vaccines that may impact CD8+ T cells. Moreover, our data highlight the importance of evaluating CD8+ T cell effector function in many infections where CD8+ T cells may be playing dual protective and pathologic roles.

## Materials and Methods

### Ethics statement

This study was conducted according to the principles specified in the Declaration of Helsinki and under local ethical guidelines (Ethical Committee of the Maternidade Climerio de Oliveira, Salvador, Bahia, Brazil; and the University of Pennsylvania Institutional Review Board). This study was approved by the Ethical Committee of the Federal University of Bahia (Salvador, Bahia, Brazil)(010/10) and the University of Pennsylvania IRB (Philadelphia, Pa)(813390). All patients provided written informed consent for the collection of samples and subsequent analysis. This study was carried out in strict accordance with the recommendations in the Guide for the Care and Use of Laboratory Animals of the National Institutes of Health. The protocol was approved by the Institutional Animal Care and Use Committee, University of Pennsylvania Animal Welfare Assurance Number A3079-01.

### Patients

All cutaneous leishmaniasis patients were seen at the health post in Corte de Pedra, Bahia, Brazil, which is a well-known area of *L. braziliensis* transmission. The criteria for diagnosis were a clinical picture characteristic of cutaneous leishmaniasis in conjunction with parasite isolation or a positive delayed-type hypersensitivity response to *Leishmania* antigen, plus histological features of cutaneous leishmaniasis. In all cases, the immunological analysis was performed before therapy. Biopsies were performed using a 4 mm punch, treated with Liberase (Roche) for 90 mins at 37°C/5% CO_2_. Biopsies were dissociated and passed through a 50 µm Medicon filter (BD phamingen). Peripheral blood mononuclear cells were obtained from heparinized venous blood layered over a Ficoll-Hypaque gradient (GE Healthcare), then washed and resuspended in RPMI1640 and stained for flow cytometry as described below.

### Microarray-based expression profiling of human lesions

For whole genome expression microarray, lesion biopsies preserved in RNAlater (Qiagen) were homogenized using a rotor-stator and RNA was isolated using the RNeasy Plus kit (Qiagen). Biotin-labeled complementary RNA (cRNA) was generated using the Illumina TotalPrep RNA amplification kit (Ambion). RNA and cRNA quality were assessed on a BioAnalyzer (Agilent). Illumina HumanHT-12 version 4 expression beadchips were hybridized with cRNA from ten *L. braziliensis* lesion biopsies and two biopses collected from uninfected donors. Data was quantile normalized and differential expression analysis was carried out using GenomeStudio v1.8 software (Illumina). Genes were considered differentially regulated if expression increased or decreased ≥3-fold with a diffscore of ≥13 or ≤−13 (equivalent to *p*≤0.05). Data was deposited on the Gene Expression Omnibus (GEO) database for public access (GSE# GSE43880). Heat map tools available on GenePattern [58] were used to graphically display differentially regulated genes in Figure 1.

### Mice

BALB/c and C57BL/6 mice (6 weeks old) were purchased from NCI, and *Rag1*−/− (B6.12957-RAG1^tm1Mom^)(N14F12), *Ifn-γ−/−* (B6.129S7-Ifng^tm1Ts^)(N8+2F23), *Prf1*−/− (perforin)(C57BL/6-Prf1^tm1Sdz^)(F?+52) were purchased from The Jackson Laboratory. C57BL/6 *IL17a*−/− mice (N9) were provided by Dr. Yoichiro Iwakura (University of Tokyo, Japan) and OT1 (B6.129S7-Rag1^tm1MomTg(TcraTcrb)^) mice were purchased from Taconic Farms and mice expressing eGFP in all T cells were originally obtained from Ulrich van Andrian (Harvard University). All mice were maintained in a specific pathogen-free environment at the University of Pennsylvania Animal Care Facilities.

### Parasites

*L. braziliensis* parasites (strain MHOM/BR/01/BA788) (deMoura et al., 2005) were grown in Schneider's insect medium (GIBCO) supplemented with 20% heat-inactivated FBS, 2 mM glutamine, 100 U/ml penicillin, and 100 µg/ml streptomycin. Metacyclic enriched promastigotes were used for infection [59]. Transgenic parasites expressing both luciferase and mCherry were generated by transfecting the parental *L. braziliensis* strain with SwaI linearized pLucCherry, a modified version of pIR1SAT encoding firefly luciferase and mCherry in the SmaI and BglII sites, respectively. Transgenic parasites were selected by plating transfected parasites on M199 agar supplemented with nourseothricin (50 µg/ml; Sigma-Aldrich). Mice were infected with 10^5^ *L. braziliensis* in the right ear, and the course of lesion progression was monitored weekly by measuring the diameter of ear induration with digital calipers (Fisher Scientific).

### Antibodies

Mouse: anti-CD45.2 APC-AlexaFluor 750, anti-CD11b eF450, anti-CD11c FITC, anti-F4/80 PE-Cy7, anti-CD3 eFluor 450, anti-IFN-γ PeCy7, anti-CD107a eFluor 660 (all from eBioscience). Anti-CD4 APC-Cy7, anti-IL-17A PE and Ly6C PerCP-Cy5.5 (BD Pharmingen), anti-CD8β PerCPCy5.5 (Biolegend) and anti-granzyme B APC (Invitrogen). Anti-Ly6G APC (Biolegend). Human: anti-CD3 APCCy7, anti-CD8a PeCy5.5 and anti-perforin FITC (all from eBioscience). Anti-CD107a PE (BD Pharmingen) and anti-granzyme B APC (Invitrogen). For in vivo CD4 or CD8 depletion, mice received i.p. injections of 250 µg of GK1.5 or 53-6.72 (BioXcell), respectively.

### Cell purification and adoptive transfer

Splenocytes from C57BL/6, *Prf1*−/−, *Ifn-γ*−/−, *Il17a*−/− and OT1 mice were collected, red blood cells lysed with ACK lysing buffer (LONZA) and CD8+ T or CD4+ T cells were purified using a magnetic bead separation kit (Miltenyi Biotec). Three million CD8+ or CD4+ T cells were transferred alone or together to *Rag1*−/− mice that were subsequently infected with *L. braziliensis*. Mice reconstituted with CD8+ T cells alone received 4 injections of 250 µg of anti-CD4 within the first 2 weeks.

### Ear preparation

Infected and uninfected ears were harvested, the dorsal and ventral layers of the ear separated, and the ears incubated in RPMI (Gibco) with 250 µg/mL of Liberase (Roche) for 90 mins at 37°C/5% CO_2_. Following incubation, the enzyme reaction was stopped using 1 mL of RPMI media containing 10% FBS. Ears were dissociated using a cell strainer (40 µm, BD Pharmingen) and an aliquot of the cell suspension was used for parasite titration.

### Parasite titration

The parasite burden in the ears was quantified as described previously [12]. Briefly, the homogenate was serially diluted (1∶10) in 96-well plates and incubated at 26°C. The number of viable parasites was calculated from the highest dilution at which parasites were observed after 7 days. To determine if parasites disseminate, footpad, opposite ear, and nose were cultured in complete Schneider's medium at 26°C and parasite growth was evaluated after 7 days.

### Flow cytometric analysis

Cell suspensions from mice were incubated with PMA (50 ng/mL), ionomycin (500 ng/mL) and Brefeldin A (10 µg/mL) (all from SIGMA) for cytokine and granzyme B intracellular staining. For degranulation assays, cells were resuspended in 4×10^6^/mL and incubated for 6 hours at 37°C/5% CO_2_ with anti-CD107a and monensin (eBioscience). Before surface and intracellular staining, cells were washed and stained for live/dead fixable aqua dead cell stain kit (Molecular Probes), according to manufacturer instructions. For human granzyme B, perforin and CD107a expression, cells were incubated for 6 hours with anti-CD107a antibody and Brefeldin A without stimulation followed by surface and intracellular staining.

### Image-stream flow cytometry

*Rag1*−/− mice were reconstituted with 3×10^6^ eGFP CD8+ T cells and infected with 10^5^ metacyclic enriched mCherry *L. braziliensis*. Six weeks post infection ear tissue was dissociated and cells were incubated with anti-CD107a fluorescent antibody for 1 hour. Total ear cell suspension was acquired on the ImageStream machine and analyzed using the IDEAS software (Amnis Corporation).

### Live cell imaging

*Rag1*−/− mice were reconstituted with 3×10^6^ eGFP CD8+ T cells and infected with 10^5^ metacyclic enriched mCherry *L. braziliensis*. Five to eight weeks post infection ears were harvested and 2×10^6^ cells used for imaging. Cells were maintained at 37°C at 5% CO_2_ on the stage of a fully enclosed microscope. Fields were selected randomly where both mCherry+ cells and eGFP+ cells could be detected. Images from multiple fields were acquired every 60 seconds on a Leica DMI 4000 inverted microscope equipped with a Yokagawa spinning disk confocal head and a Hamamatsu EMCCD 510 camera. eGFP+ and mCherry images were taken sequentially with 488 and 561 nm laser excitation, respectively. Image acquisition was controlled by MetaMorph Software.

### Histological analysis

At seven weeks post infection ears were harvested and fixed in 10% formalin. Ears were embedded in paraffin and 5 µm sections were cut and stained with hematoxylin and eosin. Histological sections were blindly scored and the system was pre-defined as the following: the lowest score (0) was defined by the absence of a lesion; moderate disease was defined as a small lesion with a localized cell infiltration ranging from minimal (1), moderate (2) to intense (3); lesions showing ulceration and intense localized cell infiltration were scored as 4; and the highest score (5) was defined by severe ulceration, cell infiltration involving the whole ear as well as cartilage destruction.

### Statistical analysis

Statistical analysis was performed with the Mann–Whitney test (two-sided t-test) using Prism (GraphPad Software).

## Supporting Information

Figure S1 **CD8+ T cells induce a greater recruitment of neutrophils in mice infected with** ***L. braziliensis*** **.** C57BL/6 and *Rag1−/−* mice were infected with *L. braziliensis* in the ear and *Rag1−/−* mice were reconstituted with either CD8+ T cells or CD8+ and CD4+ T cells or no T cells. At 7 weeks post infection mice were euthanized and cell suspensions from infected ears and contralateral ears (C57BL/6 and *Rag1−/−* only) were stained directly ex vivo for inflammatory cell markers and shown are: (A) total CD11b+ cells, (B) neutrophils, (C) macrophages, (D) monocytes and (E) dendritic cells. Representative data from three independent experiments (n = 5) with similar results are presented. *p<0.05.
(TIF)

Video S1 **CD8+ T cell moves on the surface of infected target.** Live-cell imaging of cells isolated from leishmanial lesions in *Rag1−/−* mice infected with mCherry expressing *L. braziliensis* and reconstituted with eGFP CD8+ T cells six weeks post infection. Numbers represent time in hours∶minutes∶seconds.
(MOV)

Video S2 **CD8+ T cell kills target cell and immediately detaches from target.** Live-cell imaging of cells isolated from leishmanial lesions in *Rag1−/−* mice infected with mCherry expressing *L. braziliensis* and reconstituted with eGFP CD8+ T cells four weeks post infection. Numbers represent time in hours∶minutes∶seconds.
(MOV)

Video S3 **Infected cell is killed by CD8+ T cell and loses membrane integrity.** Live-cell imaging of cells isolated from leishmanial lesions in *Rag1−/−* mice infected with mCherry expressing *L. braziliensis* and reconstituted with eGFP CD8+ T cells six weeks post infection. Numbers represent time in hours∶minutes∶seconds.
(MOV)
