# Supplementary figures and images for: Cytotoxic T Cells Mediate Pathology and Metastasis in Cutaneous Leishmaniasis
Source: PLoS Pathog. 2013 Jul 18;9(7):e1003504. doi: 10.1371/journal.ppat.1003504 (PMC3715507; doi:10.1371/journal.ppat.1003504)

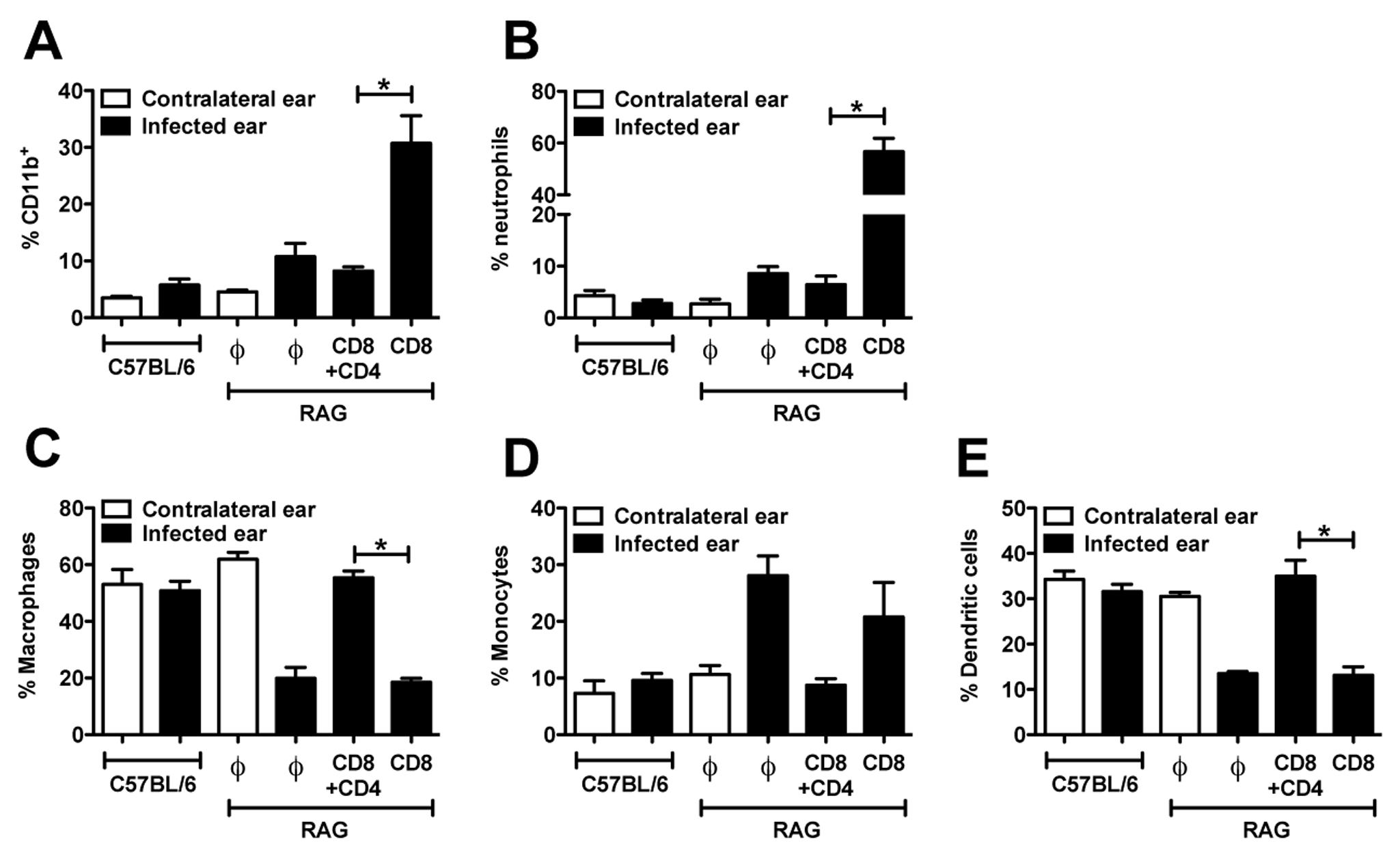

Supplement: Figure S1 — CD8+ T cells induce a greater recruitment of neutrophils in mice infected with L. braziliensis . C57BL/6 and Rag1−/− mice were infected with L. braziliensis in the ear and Rag1−/− mice were reconstituted with either CD8+ T cells or CD8+ and CD4+ T cells or no T cells. At 7 weeks post infection mice were euthanized and cell suspensions from infected ears and contralateral ears (C57BL/6 and Rag1−/− only) were stained directly ex vivo for inflammatory cell markers and shown are: (A) total CD11b+ cells, (B) neutrophils, (C) macrophages, (D) monocytes and (E) dendritic cells. Representative data from three independent experiments (n = 5) with similar results are presented. *p<0.05. (TIF) [file ppat.1003504.s001.tif]
